# Double mutant P53 (N340Q/L344R) promotes hepatocarcinogenesis through upregulation of Pim1 mediated by PKM2 and LncRNA CUDR

**DOI:** 10.18632/oncotarget.9089

**Published:** 2016-04-29

**Authors:** Mengying Wu, Jiahui An, Qidi Zheng, Xiaoru Xin, Zhuojia Lin, Xiaonan Li, Haiyan Li, Dongdong Lu

**Affiliations:** ^1^ School of Life Science and Technology, Tongji University, Shanghai, 200092, China

**Keywords:** mutant P53 (N340Q/L344R), hepatoma, PKM2, LncRNA CUDR

## Abstract

P53 is frequently mutated in human tumors as a novel gain-of-function to promote tumor development. Although dimeric (M340Q/L344R) influences on tetramerisation on site-specific post-translational modifications of p53, it is not clear how dimeric (M340Q/L344R) plays a role during hepatocarcinogenesis. Herein, we reveal that P53 (N340Q/L344R) promotes hepatocarcinogenesis through upregulation of PKM2. Mechanistically, P53 (N340Q/L344R) forms complex with CUDR and the complex binds to the promoter regions of PKM2 which enhances the expression, phosphorylation of PKM2 and its polymer formation. Thereby, the polymer PKM2 (tetramer) binds to the eleventh threonine on histone H3 that increases the phosphorylation of the eleventh threonine on histone H3 (pH3T11). Furthermore, pH3T11 blocks HDAC3 binding to H3K9Ac that prevents H3K9Ac from deacetylation and stabilizes the H3K9Ac modification. On the other hand, it also decreased tri-methylation of histone H3 on the ninth lysine (H3K9me3) and increases one methylation of histone H3 on the ninth lysine (H3K9me1). Moreover, the combination of H3K9me1 and HP1 α forms more H3K9me3-HP1α complex which binds to the promoter region of Pim1, enhancing the expression of Pim1 that enhances the expression of TERT, oncogenic lncRNA HOTAIR and reduces the TERRA expression. Ultimately, P53 (N340Q/L344R) accerlerates the growth of liver cancer cells Hep3B by activating telomerase and prolonging telomere through the cascade of P53 (N340Q/L344R)-CUDR-PKM2-pH3T11- (H3K9me1-HP1α)-Pim1- (TERT-HOTAIR-TERRA). Understanding the novel functions of P53 (N340Q/L344R) will help in the development of new liver cancer therapeutic approaches that may be useful in a broad range of cancer types.

## INTRODUCTION

Hepatocarcinogenesis is associated with many etiological factors, including hepatitis virus, AFB1, gene mutations. P53 acts as a tumor suppressor and is mutant in human cancers [1] There is growing evidence that mutant p53s promote tumor development, metastasis and progression [2, 3, 4]. For example, overexpression of mutant p53 (R273H) promotes proliferation of ovarian cancer cell [5] In addition, mutant p53 also induced platelet-derived growth factor receptor β (PDGFRβ) in human liver cancer [6] Further, the wild p53 and mutant p53 proteins show opposing functions in tumor cell motility [7] Moreover, mutant p53 can reduce dicer output [8] and blocks Smad3/N-CoR complex loading on the REGγ promoter region [9] However, the mutant p53 (Y220C) can rescue the function of unstable p53 mutants [10] Some studies indicated that activating transcription factor 3 (ATF3) bound to mutant p53 and then inhibited mutant P53 oncogenic action [11] The Myo10 controls mutant p53 functions in cancers invasion [12] and MDM2 isoforms promotes mutant p53 accumulation [13] Research shows activation of chaperone-mediated autophagy reduces the levels of accumulated mutant p53 [14] and mutant p53 proteins target micro-RNA223 expression in several cancer cells [15]. Intriguingly, mutant p53 mediates metabolic changes (e.g. glycolysis) to promote tumor progress [16, 17]. Pyruvate kinase M2 (PKM2) has been demonstrated to play a key role in metabolic regulation, gene expression, cell proliferation, cell migration, tumor angiogenesis [18, 19]. For examples, nuclear PKM2 acts as a coactivator of β-catenin to induce c-Myc expression and promotes tumorigenesis [20]

In this study, we indicate that P53 (N340Q/L344R) promotes hepatocarcinogenesis through upregulation of PKM2. PKM2 tetramer binds to the eleventh threonine on histone H3 (H3T11) that increases the phosphorylation of the eleventh lysine on histone H3 (pH3T11). Moreover, the combination of H3K9me1 and HP1α forms more H3K9me3-HP1α complex which loads onto the promoter region of Pim1, enhancing the Pim1 expression which enhances the expression of Telomerase Reverse Transcriptase (TERT), oncogenic lncRNA HOTAIR and reduces the TERRA expression.

## RESULTS

### Double mutant P53 (N340Q/L344R) facilitates liver cancer cell proliferation

**Figure 1:**
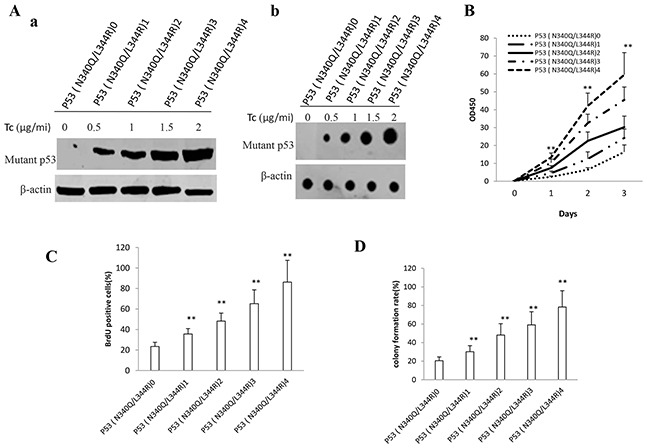
Double mutant P53 (N340Q/L344R) accelerlates liver cancer cell Hep3B growth in vitro **A.** a. The Western blotting analysis of P53 (N340Q/L344R) in stable mutant P53 (N340Q/L344R) overexpressed Hep3B cell lines infected with pLVX-Tet-On-P53 (N340Q/L344R) virus via Tet-On Advanced Inducible Expression System[tetracycline (Tc) :05-2μg/ml]. β-actin as internal control. b. The Nuclear Run on analysis of P53 (N340Q/L344R) in stable mutant P53 (N340Q/L344R) overexpressed Hep3B cell lines. β-actin as internal control. **B.** Cells growth assay using CCK8. Each value was presented as mean±standard error of the mean (SEM). **C.** S phase cells assay using BrdU. Each value was presented as mean±standard error of the mean (SEM). **D.** Cells soft agar colony formation assay. Each value was presented as mean±standard error of the mean (SEM).

To ascertain whether that double mutant P53 (N340Q/L344R) accelerlates liver cancer cell Hep3B growth *in vitro*, we first constructed the stable Hep3B cell lines (P53 null) infected with pLVX-Tet-On-P53 (N340Q/L344R) virus. P53 (N340Q/L344R) expression was confirmed by Western blotting or nuclear run on. As shown in Figure 1A (a,b), P53 (N340Q/L344R) was overexpressed in Tet on Hep3B cell lines and the expression was the highest when the tetracycline (Tc) was added up to 2μg/ml. At the first time, we detected these cells proliferation in vitro. As shown in Figure 1B, P53 (N340Q/L344R) overexpression promoted the Hep3B cell proliferation ability compared to control (P<0.01). Notably, the cell grow rate reached maximum when the Tc concentration was 2μg/ml. Next, we detected the S phase cells by BrdU staining in P53 (N340Q/L344R) overexpression Hep3B. The BrdU staining findings showed that the BrdU positive rate added up to 23.4%,35.6%,48.2%,%,65.2%,86.2% in P53 (N340Q/L344R) overexpressed Hep3B when Tc concentration was 0,0.5,1,1.5,2.0μg/ml respectively (P<0.01) (Figure 1C). Then we conducted soft-agar colony-formation efficiency assay in these Hep3B cells. The soft-agar colony-formation rate added up to 20.5%,30.2%,48.1%,%,59.1%,78.3% when Tc concentration was 0,0.5,1,1.5,2.0μg/ml respectively (P<0.01) (Figure 1D). Taken together, these observations suggest that double mutant P53 (N340Q/L344R) promotes liver cancer cells proliferation.

### Double mutant P53 (N340Q/L344R) accelerates liver cancer cell growth *in vivo*

**Figure 2:**
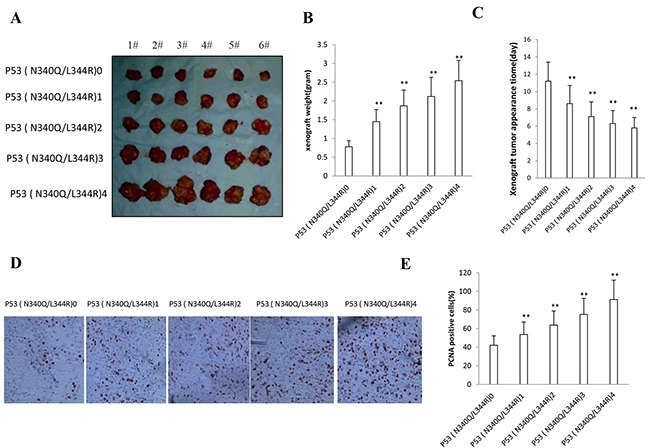
Double mutant P53 (N340Q/L344R) promotes liver cancer cell Hep3B growth *in vivo* **A.** The mice were stratified and the tumors were recovered. The photography of xenograft tumors in the five groups (indicated in left). **B.** The wet weight of each tumor was determined for each mouse. Each value was presented as mean±standard error of the mean (SEM). **C.** The Xenograft appearance time (days). Each value was presented as mean±standard error of the mean (SEM). **D.** A portion of each tumor was fixed in 4% paraformaldehyde and embedded in paraffin for anti-PCNA immunostainning. (original magnification×100). **E.** PCNA positive cells analysis. Each value was presented as mean±standard error of the mean (SEM).

To explore whether mutant P53 (N340Q/L344R) promotes liver cancer cell growth *in vivo*, the Hep3B stable cell lines with different expression of mutant P53 (N340Q/L344R) were injected subcutaneously into Balb/C (severe combined immunodeficiency) mice. As shown in Figure 2A & 2B, when mutant P53 (N340Q/L344R) was overexpressed at the Tc Con.0,0.5,1,1.5,2.0μg/ml respectively, the xenograft tumor weight increased approximately 1.5,2,2.5,3 folds when compared to the corresponding control group respectively (1.45grams, 1.87grams, 2.12grams, 2.54grams versus 0.78grams, P<0.01). Mutant P53 (N340Q/L344R) overexpression resulted in early xenograft tumor formation compared to the control group at the Tc Con.0,0.5,1,1.5,2μg/ml respectively (8.6days, 7.1days, 6.3days, 5.8days versus 11.2 days, P<0.05) (Figure 2C). Xenograft tumor tissue possessed more poor-differentiation cells in mutant P53 (N340Q/L344R) overexpression group than that of control group, suggesting that mutant P53 (N340Q/L344R) overexpression enhanced the xenografts tumor malignant grade. The proliferation index was significantly higher in mutant P53 (N340Q/L344R) overexpressed tumors compared to the control at the Tc Con.0,0.5,1,1.5,2.0μg/ml respectively (53.6%, 63.7%, 75.2%,91.2% versus 42.1%, P<0.01) (Figure 2D & 2E). Collectively, these findings demonstrate that mutant P53 (N340Q/L344R) enhances liver cancer progression *in vivo*.

### Mutant P53 (N340Q/L344R) enhances PKM2 expression and its polymer formation

**Figure 3:**
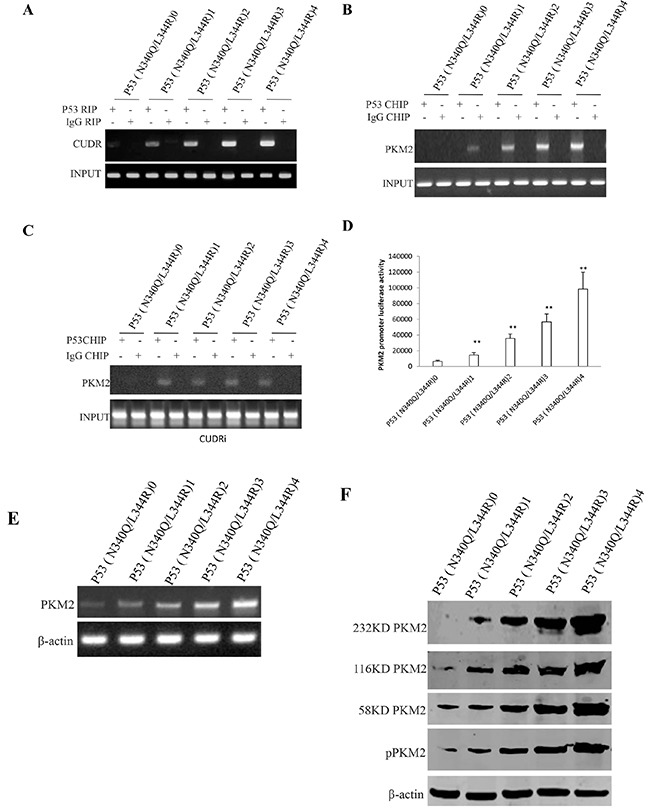
mutant P53 (N340Q/L344R) enhances PKM2 expression dependent on SPONGE CUDR **A.** RNA Immunoprecipitation (RIP) with anti-P53 (N340Q/L344R) followed by RT-PCR with CUDR mRNA primers in P53 (N340Q/L344R) overexpressed Hep3B cell lines infected with pLVX-Tet-On-P53 (N340Q/L344R) virus via Tet-On Advanced Inducible Expression System[tetracycline (Tc) :05-2μg/ml]. IgG RIP as negative control. CUDR mRNA as INPUT. **B.** Chromatin Immunoprecipitation (CHIP) with anti- anti-P53 (N340Q/L344R) followed by PCR with PKM2 promoter primers in in P53 (N340Q/L344R) overexpressed Hep3B cell lines. IgG CHIP as negative control. PMK2promoter DNA as INPUT. **C.** Chromatin Immunoprecipitation (CHIP) with anti- anti-P53 (N340Q/L344R) followed by PCR with PKM2 promoter primers in in P53 (N340Q/L344R) overexpressed and CUDR knocked-down Hep3B cell lines. IgG CHIP as negative control. PMK2 promoter DNA as INPUT. **D.** PKM2 promoter luciferase activity assay in mutant P53 (N340Q/L344R) overexpressed Hep3B cell lines. Each value was presented as mean±standard error of the mean (SEM). **E.** PKM2 expression analysis by RT-PCR in mutant P53 (N340Q/L344R) overexpressed Hep3B cell lines. β-actin as internal control. **F.** Western blotting with anti-PKM2 and anti-pPKM2 in mutant P53 (N340Q/L344R) overexpressed Hep3B cell lines. β-actin as internal control.

To identify whether mutant P53 (N340Q/L344R) could alter PKM2 turnout and its activity, we performed RNA Immunoprecipitation (RIP), Chromatin Immunoprecipitation (CHIP), promoter luciferase activity assay, RT-PCR and Western blotting in Hep3B cell lines. As shown in Figure 3A, mutant P53 (N340Q/L344R) overexpression gradually increased the interaction between mutant P53 (N340Q/L344R) and noncoding RNA CUDR when the tetracycline (Tc) was added from 0.5mg/L to 2mg/L. Chromatin Immunoprecipitation (CHIP) results showed that mutant P53 (N340Q/L344R) overexpression gradually increased the mutant P53 (N340Q/L344R loading onto the PKM2 promoter region when the tetracycline (Tc) was added from 0.5μg/ml to 2μg/ml (Figure 3B). Intriguingly, the action was abrogated when CUDR was knocked down (Figure 3C). The luciferase activity assay showed that mutant P53 (N340Q/L344R) overexpression gradually increased the PKM2 promoter luciferase activity when the tetracycline (Tc) was added from 0.5mg/L to 2mg/L (Figure 3D). RT-PCR results showed that P53 (N340Q/L344R) overexpression gradually increased the PKM2 transcription when the tetracycline (Tc) was added from 0.5mg/L to 2mg/L (Figure 3E). Western blotting results showed that P53 (N340Q/L344R) overexpression gradually increased the PKM2 expression and its dimer (PKM2-PKM2), trimer (PKM2-PKM2-PKM2-PKM2) and polymer formation (PKM2)_n_ when the tetracycline (Tc) was added from 0.5mg/L to 2mg/L (Figure 3F). Takentogether, these observations suggest that mutant P53 (N340Q/L344R) enhances PKM2 expression and its polymer formation.

### Mutant P53 (N340Q/L344R) promotes the interplay between H3K9me1 and HP1α

**Figure 4:**
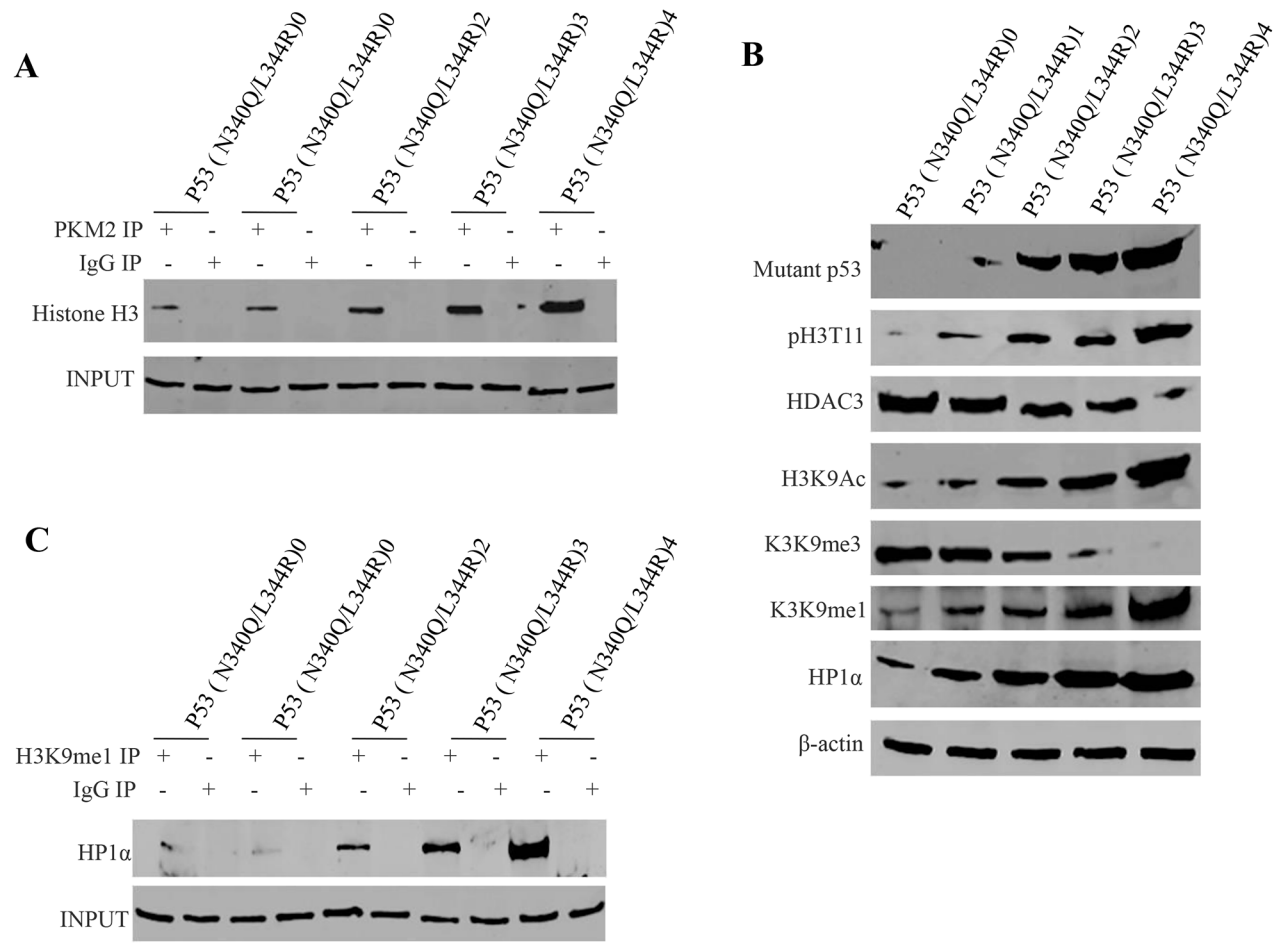
mutant P53 (N340Q/L344R) promotes the interplay between H3K9me1 and HP1α **A.** anti-PKM2 Co-Immunoprecipitation (IP) followed by Western blotting with Histone H3 in P53 (N340Q/L344R) overexpressed Hep3B cell lines infected with pLVX-Tet-On-P53 (N340Q/L344R) virus via Tet-On Advanced Inducible Expression System[tetracycline (Tc) :05-2.0μg/ml]. IgG IP as negative control. INPUT refers to Western blotting with anti-PKM2. **B.** Western blotting with anti-P53 (N340Q/L344R), anti-pH3K11, anti-HDAC3, anti-H3K9Ac, anti-H3K9me3, anti-H3K9me1, anti-HP1α.β-actin as internal control. **C.** anti-H3K9me1 Co-Immunoprecipitation (IP) followed by Western blotting with anti-HP1α in P53 (N340Q/L344R) overexpressed Hep3B cell lines infected with pLVX-Tet-On-P53 (N340Q/L344R) virus via Tet-On Advanced Inducible Expression System[tetracycline (Tc):05-2μg/ml]. IgG IP as negative control. INPUT refers to Western blotting with anti-H3K9me1.

To address whether PKM2 impact on Mutant P53 (N340/L344R) functions, we first prefromed Co-Immunoprecipitation (IP) with anti-PKM2. As showed in Figure 4A, mutant P53 (N340Q/L344R) overexpression gradually increased the interaction between PKM2 and Histone H3 when the tetracycline (Tc) was added from 0.5mg/L to 2mg/L. Intriguingly, mutant P53 (N340Q/L344R) overexpression gradually increased histone phosphorylation modification on H3T11 which caused the increment of H3K9Ac, H3K9me and HP1α expression, and the decrease of the H3K9me3 and HDAC3 (Figure 4B). On the other hand, Co-immunoprecipitationresults showed that mutant P53 (N340Q/L344R) overexpression gradually increasedthe interaction between H3K9me and HP1α when the tetracycline (Tc) was added from 0.5mg/L to 2mg/L (Figure 4C). Taken together, Mutant P53 (N340Q/L344R) promotes the interplay between H3K9me1 and HP1α due to the PKM2 increment and activation.

### Mutant P53 (N340Q/L344R) enhances Pim1 expression triggered by the increased interplay between H3K9me1 and HP1α

**Figure 5:**
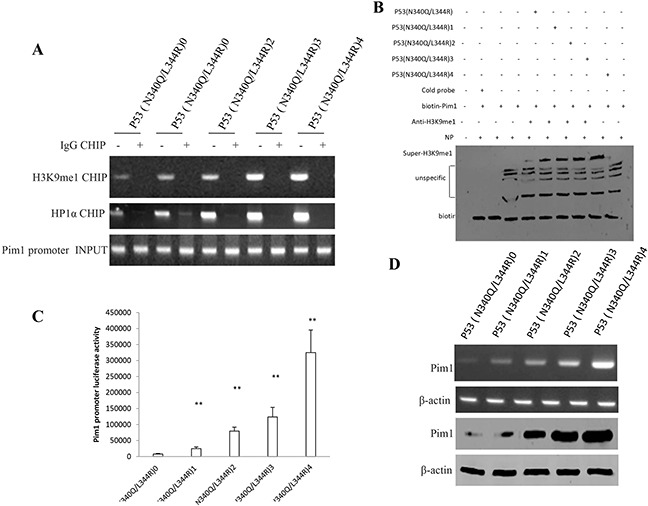
Mutant P53 (N340Q/L344R) enhances Pim1 expression triggered by the increased interplay between H3K9me1 and HP1α **A.** Chromatin Immunoprecipitation (CHIP) with anti-H3K9me1, anti-HP1α followed by PCR with Pim1 promoter primers anti-Histone in P53 (N340Q/L344R) overexpressed Hep3B cell lines infected with pLVX-Tet-On-P53 (N340Q/L344R) virus via Tet-On Advanced Inducible Expression System[tetracycline (Tc) :05-2μg/ml]. IgG CHIP as negative control. Pim1 promoter as INPUT. **B.** Super-EMSA (gel-shift) with biotin- pim1 promoter probe and anti-H3k9me1 antibody. The intensity of the band was examined by Western blotting with anti-Bioton. **C.** Pim1 promoter luciferase activity assay in mutant P53 (N340Q/L344R) overexpressed HepG2 cell lines. Each value was presented as mean±standard error of the mean (SEM). **D.** pim1 expression analysis by RT-PCR (upper) and Western blotting (lower) with anti-Pim1 in mutant P53 (N340Q/L344R) overexpressed HepG2 cell lines. β-actin as internal control.

To prove whether Mutant P53 (N340Q/L344R) enhanced the Pim1 expression in liver cancer cells Hep3B, we first prefromed Chromatin -Immunoprecipitation (CHIP) with anti-H3K9me1 and anti-HP1α. As showed in Figure 5A, mutant P53 (N340Q/L344R) overexpression gradually increased the loading of H3K9me1 and HP1α on Pim1 promoter when the tetracycline (Tc) was added from 0.5μg/ml to 2μg/ml. Super-EMSA results showed that mutant P53 (N340Q/L344R) overexpression gradually increased the interaction between H3K9me1 and Pim1 promoter probe (Figure 5B). Pim1 promoter luciferase activity assay showed that mutant P53 (N340Q/L344R) overexpression gradually increased pim1 promoter luciferase activity when the tetracycline (Tc) was added from 0.5mg/L to 2mg/L. (Figure 5C). Mutant P53 (N340Q/L344R) overexpression gradually increased Pim1 expression when the tetracycline (Tc) was added from 0.5mg/L to 2mg/L. (Figure 5D). Collectively, mutant P53 (N340Q/L344R) enhances Pim1 expression triggered by the increased interplay between H3K9me1 and HP1α.

### Mutant P53 (N340Q/L344R) enhances HOTAIR expression and stimulates telomerase activity dependent on pim1

**Figure 6:**
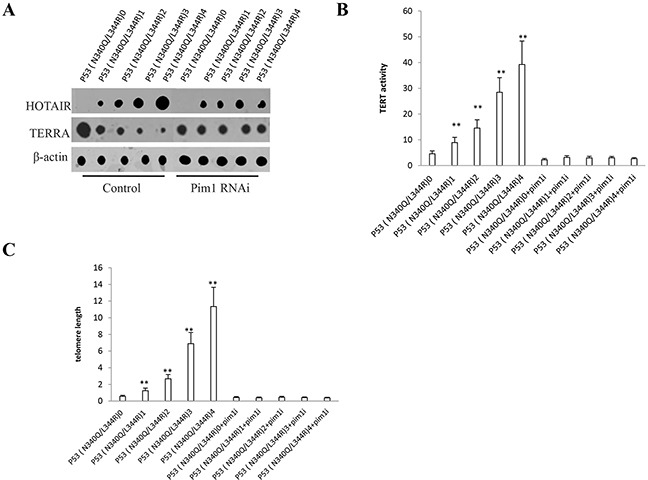
mutant P53 (N340Q/L344R) enhances HOTAIR expression and stimulates telomerase activity through pim1 **A.** Nuclear run on assay for HOTAIR and TERRA in P53 (N340Q/L344R) overexpressed Hep3B cell lines infected with pLVX-Tet-On-P53 (N340Q/L344R) virus via Tet-On Advanced Inducible Expression System[tetracycline (Tc) :05-2μg/ml]. β-actin as internal control. **B.** The telomerase activity was measured by using Quantitative Telomerase Detection. Each value was presented as mean±standard error of the mean (SEM). **C.** The telomere length was measured by using Quantitative PCR. Each value was presented as mean±standard error of the mean (SEM).

Given that P53 (N340Q/L344R) promoted the Pim1 activation, we have a reason to consider whether P53 (N340Q/L344R) couldinfluences on key oncogene via pim1. In P53 (N340Q/L344R) overexpressed Hep3B cell lines infected with pLVX-Tet-On-P53 (N340Q/L344R) virus via Tet-On Advanced Inducible Expression System[tetracycline (Tc), we found P53 (N340Q/L344R) overexpression gradually increased lncRNA HOTAIR expression and gradually decreased the TERRA expression when the tetracycline (Tc) was added from 0.5μg/ml to 2μg/ml. However, the action was abrogated when pim1 was knocked down (Figure 6A). The telomerase activity was measured by using Quantitative Telomerase Detection and the results showed that P53 (N340Q/L344R) overexpression gradually increased the telomerase activity when the tetracycline (Tc) was added from 0.5μg/ml to 2μg/ml (Figure 6B). The telomere length was measured byusingQuantitative PCR and P53 (N340Q/L344R) overexpression gradually increased the telomere length when the tetracycline (Tc) was added from 0.5μg/ml to 2μg/ml. However, the action was abrogated when pim1 was knocked down (Figure 6C). Collectively, these observations suggest that mutant P53 (N340Q/L344R) enhances HOTAIR expression and stimulates telomerase activity through pim1.

### Depletion of PKM2 abrogated the mutant P53 (N340Q/L344R) oncogenic function

**Figure 7:**
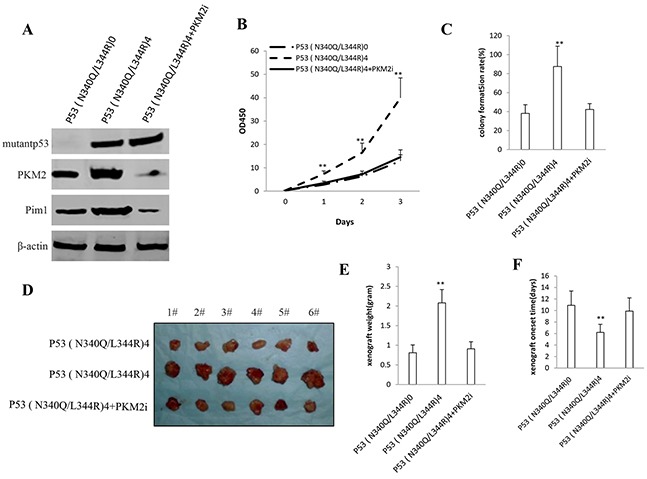
The rescued experiment of carcinogenesis effect of the mutant P53 (N340Q/L344R) PKM2 knockdown abrogated the Mutant P53 (N340Q/L344R) oncogenic function in Hep3B cell lines infected with control plasmid, pLVX-Tet-On-P53 (N340Q/L344R) [Tc: 2μg/ml], pLVX-Tet-On-P53 (N340Q/L344R) plus pGFP-V-RS-PKM2. **A.** The western blotting analysis with anti-P53 (fulllength) and anti-PKM2.β -actin as internal control. **B.** Cells growth assay using CCK8. Each value was presented as mean±standard error of the mean (SEM). **C.** Cells soft agar colony formation assay. Each value was presented as mean±standard error of the mean (SEM). **D.** In vivo test in Hep3B cell lines infected with control plasmid, pLVX-Tet-On-P53 (N340Q/L344R) [Tc: 2μg/ml], pLVX-Tet-On-P53 (N340Q/L344R) plus pGFP-V-RS-PKM2. The mice were stratified and the tumors were recovered. The photography of xerograft tumor in the three groups (indicated in left). **E.** The wet weight of each tumor was determined for each mouse. Each value was presented as mean±standard error of the mean (SEM). **F.** The Xenograft appearance time. Each value was presented as mean±standard error of the mean (SEM).

Given that mutant P53 (N340Q/L344R) promotes hepatocarcinogenesis via PKM2, we further transfected pGFP-V-RS-PKM2 into the cells (PKM2 RNAi) in Hep3Bcell lines infected with control plasmid, pLVX-Tet-On-P53 (N340Q/L344R) [Tc: 2μg/ml]). As shown in Figure 7A, the western blotting analysis with anti-P53 and anti-PKM2 showed that mutantP53 (N340Q/L344R) was overexpressed and PKM2 knocked down. and pim1 expression was increased in P53 (N340Q/L344R) overexpressed Hep3B cells and decreased in P53 (N340Q/L344R) overexpressed plus PKM2 knocked down Hep3B cells. mutantP53 (N340Q/L344R) promotes Hep3B cell proliferation, while the action was abrogated in P53 (N340Q/L344R) overexpressed plus PKM2 knocked down Hep3B cells (Figure 7B). mutantP53 (N340Q/L344R) promotes Hep3B cell colony formation ability (87.5%versus 38.1%, P<0.01), as well as the action was abrogated in P53 (N340Q/L344R) overexpressed plus PKM2 knocked down Hep3B cells (42.2% versus 38.1%, P>0.05) (Figure 7C). In tumorigenesis *in vivo* test, the xenografts tumors weights were significantly increased in mutantP53 (N340Q/L344R) overexpressed Hep3B cell compared to P53 (N340Q/L344R) overexpressed plus PKM2 knocked down Hep3B cells (2.08 gram versus 0.81 gram, P<0.01), as well as the action was abrogated in P53 (N340Q/L344R) overexpressed plus PKM2 knocked-down Hep3B cells (0.91gram versus 0.81 gram, P>0.05) (Figure 7D, 7E). The xenografts tumors onset time was significantly shorten in mutantP53 (N340Q/L344R) overexpressed Hep3B cell compared to P53 (N340Q/L344R) overexpressed plus PKM2 knocked down Hep3B cells (6.2 days versus 10.9 days, P<0.01), as well as the action was abrogated in P53 (N340Q/L344R) overexpressed plus PKM2 knocked down Hep3B cells (9.9days versus 10.9 days, P>0.05) (Figure 7F). Together, these results suggest depletion of PKM2 abrogated the Mutant P53 (N340Q/L344R) oncogenic function.

## DISCUSSION

**Figure 8:**
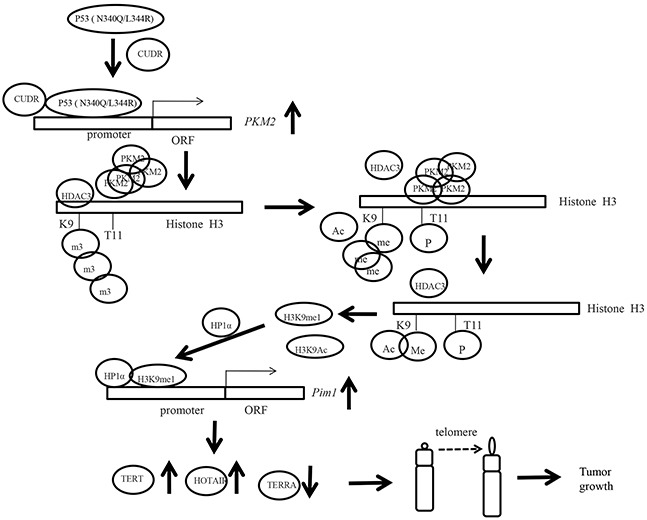
The schematic diagram illustrates a model that P53 (N340Q/L344R) promotes hepatocarcinogenesis through upregulation of PKM2 Both P53 (N340Q/L344R) and PKM2 are upregulated in human hepatocellular carcinoma tissues, and present the positive correlation. And the P53 (N340Q/L344R) promotes the growth of hepatocellular carcinoma cells in vitro and in vivo. Mechanistically, P53 (N340Q/L344R) forms complex with CUDR and the complex binds to the promoter regions of PKM2 which enhances the expression, phosphorylation of PKM2 and its polymer formation. Thereby, the polymer PKM2 (tetramer) binds to the eleventh threonine on histone H3 that increases the phosphorylation of the eleventh lysine on histone H3 (pH3T11). Furthermore, pH3T11 blocks HDAC3 binding to H3K9Ac that prevents H3K9Ac from deacetylation and stabilizes the H3K9Ac modification. On the other hand, it also decreased tri-methylation of the ninth lysine ninth on histone3 (H3K9me3) and increases one methylation of the ninth lysine ninth on histone H3 (H3K9me1). Moreover, the combination of H3K9me1 and HP1 α forms more H3K9me3-HP1α complex. This increased complex binds to the promoter region of Pim1, enhancing the expression of Pim1 that increases the expression of TERT, oncogenic lncRNA HOTAIR and reduced expression of TERRA expression. Ultimately, P53 (N340Q/L344R) accerlerates the growth of hepatocellular carcinoma cells by activated telomerase and prolonged telomere through the cascade of P53 (N340Q/L344R)-CUDR-PKM2-pH3T11- (H3K9me1-HP1α)-Pim1- (TERT-HOTAIR-TERRA).

To this data, mutant P53 (N340Q/L344R) shows a strong oncogenic function mediated by PKM2 (Figure 8). P53 (N340Q/L344R) promotes hepatocarcinogenesis through upregulation of PKM2. Both P53 (N340Q/L344R) and PKM2 are upregulated in human hepatocellular carcinoma tissues, and present the positive correlation. And the P53 (N340Q/L344R) promotes the liver cancer cell's growth. Mechanistically, P53 (N340Q/L344R) forms complex with CUDR and the complex binds to the promoter regions of PKM2 which enhances the expression, phosphorylation of PKM2 and its polymer formation. Thereby, the polymer PKM2 (tetramer) binds to the eleventh serine on histone H3 that increases the phosphorylation of the eleventh threonine on histone H3 (pH3T11). Furthermore, pH3T11 blocks HDAC3 binding to H3K9Ac that prevents H3K9Ac from deacetylation and stabilizes the H3K9Ac modification. On the other hand, it also decreased tri-methylation of the ninth lysine ninth on histone3 (H3K9me3) and increases one methylation of the ninth lysine ninth on histone H3 (H3K9me1). Moreover, the combination of H3K9me1 and HP1 α forms more H3K9me3-HP1α complex which binds to the promoter region of Pim1, enhancing the expression of Pim1 that enhances the expression of TERT, oncogenic lncRNA HOTAIR and reduces the TERRA expression. Ultimately, P53 (N340Q/L344R) accerlerates the growth of hepatocellular carcinoma cells by activated telomerase and prolonged telomere through the cascade of P53 (N340Q/L344R) -CUDR-PKM2-pH3T11- (H3K9me1-HP1α)-Pim1- (TERT-HOTAIR-TERRA).

It is worth mentioning that double mutant P53 (N340Q/L344R) may play an important role in the occurrence of liver cancer cancer. In this report, we focused mainly on the view how double mutant P53 (N340Q/L344R) functions during hepatocarcinogenesis. Although dimeric (M340Q/L344R) influences on the post-translational modifications of p53, it is not clear how dimeric (M340Q/L344R) plays a role during hepatocarcinogenesis [21]. Our results indicates that P53 (N340Q/L344R) has a strong oncogenic charter. To this date, accumulating evidence indicates mutant P53 function in liver cancer. A large number of modifications on p53 (e.g. 3KR mutant) regulate cellular DNA binding ability and tumor development [22, 23, 24]. Moreover, several p53 mutant proteins escape proteolytic degradation and exert oncogenic gain-of-function properties [25, 26] or causes maintenance of genomic integrity [27, 28, 30] Intruigingly, mutant P53 facilitates dedifferentiation of mature hepatocytes into progenitor-like cells [29] Our present findings are consistent with some reports. Herein, the involvement of promotion of liver cancer cells growth based on double mutant P53 (N340Q/L344R) is supported by results from two parallel sets of experiments: (1) double mutant P53 (N340Q/L344R) facilitates liver cancer cell proliferation. (2)double mutant P53 (N340Q/L344R) accelerates liver cancer cell growth *in vivo.*

It has been confirmed that PKM2 plays a role in tumor anabolic metabolism [30] For example, knockdown of PKM2 suppressed aerobic glycolysis of liver cancer cell [31] In this study, we found that double mutant P53 (N340Q/L344R) enhanced the pyruvate kinase M2 isoform (PKM2) activity. In fact, PKM2 is required for cell survival and proliferation in tumorigenesis [32, 33] Moreover, the PKM2 dimers and tetramers are critical for tumorigenesis and epithelial-mesenchymal transition which is controlled by multiple factors, e.g. miR675 and H19 [34, 37] It is worth noting that our findings in this study provide novel evidence for an active role of PKM2 based on double mutant P53 (N340Q/L344R) in liver cancer cell growth. This assertion is based on several observations: (1)Mutant P53 (N340Q/L344R) enhances PKM2 expression and its polymer formation. (2) Depletion of PKM2 abrogated the double Mutant P53 (N340Q/L344R) oncogenic function.

Strikingly, our results demonstrates that P53 (N340Q/L344R) forms complex with CUDR and the complex binds to the promoter regions of PKM2 which enhances the expression, phosphorylation of PKM2 and its polymer formation. In fact, CUDR is closely associated with tumorigenesis. For example, CUDR increased the exprseeion of HULC, β-Catenin, TERT and C-myc in human liver cancer stem cell [36, 37]. Moreover, CUDR cooperates with SET1A to trigger stem cell malignant transformation [38].

Of significance, our findings show that double mutant P53 (N340Q/L344R) promotes pim1 expression through H3K9me1 and HP1α dependent on PKM2, and enhances HOTAIR expression, telomerase activity, elongates telomere length in liver cancer cells. This assertion is based on several observations (1)double mutant P53 (N340Q/L344R) promotes the interplay between H3K9me1 and HP1α. (2)Mutant P53 (N340Q/L344R) enhances Pim1 expression triggered by the increased interplay between H3K9me1 and HP1α. (3)double mutant P53 (N340Q/L344R) enhances HOTAIR expression and stimulates telomerase activity dependent on pim1. Pim-1 is associated with transcriptional activation [39]. Our previous studies showed that H19 enhanced Pim1 expression and function in the liver cancer [34]. Telomeric repeat-containing RNA (TERRA) is important for telomere regulation [40] and TRF2 represses TERRA transcription through its homodimerization domain [41]. Our previous study showed that the overexpression of H19 increases the binding of TERT to TERC and reduces the interplay between TERT with TERRA, thus enhancing the cell telomerase activity and extending the telomere length in liver cancer stem cells [37]. HOTAIR is is reported to reprogram chromatin organization and promote tumor progression and metastasis [42, 43, 44]. For examples, HOTAIR could promote migration and invasion of hepatocellular carcinoma (HCC) cells and promotes human liver cancer stem cell malignant growth [45, 46]. In addition, HOTAIR preferentially occupies a GA-rich DNA motif to nucleate broad domains of polycomb occupancy [47] and altered histone H3 lysine 27 methylation, gene expression, and increased cancer invasiveness [48].

We should further explore the function of duble mutant P53 (N340Q/L344R) during hepatocarcinogenesis. For example, what causes strong oncogenic action duble mutant P53 (N340Q/L344R)? How does duble mutant P53 (N340Q/L344R) do? Does CUDR regulate a series of molecular signaling pathway in liver cancer cells? Answering these questions will help understand the mechanism about hepatocarcinogenesis. Duble mutant P53 (N340Q/L344R) accerlerates the growth of hepatocellular carcinoma cells by activated telomerase and prolonged telomere through the cascade of P53 (N340Q/L344R)-CUDR-PKM2-pH3T11−(H3K9me1-HP1α)-Pim1−(TERT− HOTAIR- TERRA). Understanding the functions of mutant p53 will help in the development of new therapeutic approaches that may be useful in a broad range of cancer types [49].

## MATERIALS AND METHODS

### Cell lines and plasmids

Human hepatoma cell lines Hep3B were obtained from the Cell Bank of Chinese Academy of Sciences (Shanghai, China). These cell lines were maintained in Dulbecco's modified Eagle medium (Gibco BRL Life Technologies) supplemented with 10% heat-inactivated fetal bovine serum (sigma) in a humidified atmosphere of 5% CO_2_ incubator at 37°C. pLVX-Tet-On-Advanced vector and pVSVG were purchased from Cloneth. pVSVG-P53 (N340Q/L344R), PGFP-V-RS-PKM2, pGFP-V-RS-Pim1 were prepared by ourselves.

### Cell transfection and stable cell lines

Cells were transfected with DNA plasmids using transfast transfection reagent lipofectamine^R^ 2000 (Invitrogen) according to manufacturer's instructions. For screening stable cell lines, forty-eight hours after transfection, cells were plated in the selective medium containing G418 (2000μg/ml, Invitrogen) for the next 4 weeks or so, and the selective media were replaced every 3 days.

### Selection of ShRNA cell lines

Cell lines were transfected with pGFP-V-RS-PKM2, pGFP-V-RS-Pim1 using transfection reagent lipofectamine^R^ 2000 (Invitrogen). Forty-eight hours after transfection, the cells were cultured with the selection media containing 2 μg/ml Puromycin (Invitrogen) for PKM2 or Pim1 knockdown. The selection media were replaced every 3 days.

### Tet-On advanced inducible expression

pLVX-Tet-On Advanced System was performed according to the manufacturer's instructions. As to the first stable transfection, the Hep3B cells were transfected with pLVX-Tet-On vector (Clonth) using Lipofecamine^TM^2000 (Invitrogen), and then established the stable cell line by selecting and screening using 2mg/ml G418 (BD Biosciences Clontech). To ensure optimal induction and low background, multiple clonal cell lines must be screened after each stable transfection using western blotting. As to the Second stable transfection, pVSVG/ P53 (N340Q/L344R was infected into the pLVX-Tet-On Hep3B stable cell line, and transfected cells were grown in presence of 0.05 mg/ml Zeo (BD Biosciences Clontech) for selection. Individual colonies were isolated and screened for P53 (N340Q/L344R expression. The positive clones were cultured in DMEM medium (Gibco BRL Life Technologies) containing the indicated tetracycline (Tc) (sigma) concentrations (0-2μg/ml) to induce the P53 (N340Q/L344R expression.

### RT-PCR

Total RNA was purified using Trizol (Invitrogen) according to manufacturer's instructions. cDNA was prepared by using oligonucleotide (dT)_17-18_, random primers, and a SuperScript First-Strand Synthesis System (Invitrogen). PCR analysis was performed under the specical conditions. β-actin was used as an internal control.

### Western blotting

The logarithmically growing cells were washed twice with ice-cold phosphate-buffered saline (PBS, Hyclone) and lysed in a RIPA lysis buffer. Cells lysates were centrifuged at 12,000g for 20 minutes at 4°C after sonication on ice, and the supernatant were separated. After being boiled for 5-10 minutes in the presence of 2-mercaptoethanol, samples containing cells proteins were separated on a 10% sodium dodecyl sulfate-polyacrylamide gel electrophoresis (SDS-PAGE) and transferred onto a nitrocellulose membranes (Invitrogen, Carlsbad, CA, USA). Then blocked in 10% dry milk-TBST (20mM Tris-HCl [PH 7.6], 127mM NaCl, 0.1% Tween 20) for 1 h at 37°C. Following three washes in Tris-HCl pH 7.5 with 0.1% Tween 20, the blots were incubated with 0.2 μg/ml of antibody (appropriate dilution) overnight at 4°C. Following three washes, membranes were then incubated with secondary antibody for 60 min at 37°C or 4°C overnight in TBST. Signals were visualized by ODYSSEY infrared imaging system (LI-COR, Lincoln, Nebraska USA). IRDye 680LT /IRDye 800CW secondary antibodies were purchased from LI-COR scientific company.

### Co-immunoprecipitation (IP)

Cells were lysed in 1 ml of the whole-cell extract buffer A (50mM pH7.6 Tris-HCl, 150mMNaCl, 1%NP40, 0.1mMEDTA,1.0mM DTT,0.2mMPMSF, 0.1mM Pepstatine,0.1mM Leupeptine,0.1mM Aproine). Five-hundred-microliter cell lysates was used in immunoprecipitation with antibody. In brief, protein was pre-cleared with 30μl protein G/A-plus agarose beads (Santa Cruz, Biotechnology, Inc. CA) for 1 hour at 4°C and the supernatant was obtained after centrifugation (5,000rpm) at 4°C. Precleared homogenates (supernatant) were incubated with 2 μg of antibody and/or normal mouse/rabbit IgG by rotation for 4 hours at 4°C, and then the immunoprecipitates were incubated with 30μl protein G/A-plus agarose beads by rotation overnight at 4°C, and then centrifuged at 5000rpm for 5 min at 4°C. The precipitates were washed five times×10min with beads wash solution (50 mM pH7.6 TrisCl,150mMNaCl,0.1%NP-40,1mM EDTA) and then resuspended in 60μl 2×SDS-PAGE sample loading buffer to incubate for 10 min at 100°C. Then Western blot was performed with a another related antibody indicated in Western blotting.

### RNA immunoprecipitation (RIP)

Cells were lysed (15 min, 4°C) in 100 mM KCl, 5 mM MgCl_2_, 10 mM HEPES [pH 7.0], 0.5% NP40, 1 mM DTT, 100 units/ml RNase OUT (Invitrogen), 400 μM vanadyl-ribonucleoside complex and protease inhibitors (Roche). The lysates were incubated with specific antibody or normal mouse/rabbit IgG overnight at 4°C, followed that the lysates were incubated with protein A/G-plus agarose beads (Santa Cruz, Biotechnology, Inc. CA) 4 hours at 4°C. Then the beads were subsequently washed four times with 50 mM Tris-HCl (pH 7.0), 150 mM NaCl, 1 mM MgCl_2_, and 0.05% NP-40, and twice after addition of 1M Urea. RNA is isolated from the Immunoprecipitates (IPs) and RT-PCR is performed with the primers as follows: CUDR/P1:5′-atgagtcccatcatctctcca-3′; CUDR/P2: 5′-taatgtaggtggcgatgagtt-3′.

### Chromatin immunoprecipitation (CHIP) assay

Cells were cross-linked with 1% (v/v) formaldehyde (Sigma) for 10 min at room temperature and stopped with 125 mm glycine for 5 min. Crossed-linked cells were washed with phosphate-buffered saline, resuspended in lysis buffer, and sonicated for 8-10 min in a SONICS VibraCell to generate DNA fragments with an average size of 500 bp or so. Chromatin extracts were diluted 5-fold with dilution buffer, pre-cleared with Protein-A/G-Sepharose beads, and immunoprecipitated with specific antibody on Protein-A/G-Sepharose beads. After washing, elution and de-cross-linking, the ChIP DNA was detected by either traditional PCR.

### Super-EMSA (Gel-shift)

Cells were washed and scraped in ice-cold PBS to prepare nuclei for electrophoretic gel mobility shift assay with the use of the gel shift assay system modified according to the manufacturer's instructions (Promega). In brief, consensus oligonucleotides for damage or repair DNA was biotin-labeled (hot probe). Each binding reaction was carried out with 1 μg biotinylated dsDNA probe and 200 μg purified nuclear protein in 20 μl of binding buffer containing 0.5mg/ml poly (dI:dC) (25 mM HEPES at PH8.0 with 50 mM KCl. 0.1% Triton X100, 2 mM MgCl2, 3 mM DTT, and 5% glycerol). Twenty-five pmol unlabeled cold DNA motifs (a 250-fold excess) were added in the competition assays. Reactions were carried out for 30 min incubation at room temperature, followed by overnight incubation at 4°C. Reaction mixtures were loaded onto 6% TBE polyacrylamide gels and separated in 0.5×TBE at 100v on ice until the dye front migrated two-thirds of the way to NC membranes and Western blotting for anti-biotin.

### Dual luciferase reporter assay

Cells (1×10^5^/well of a six-well plate) were transiently transfected by use of the Lipofectiamine^TM^ 2000 (Invitrogen). After incubation for 48 h, the cells were harvested with Passive Lysis Buffer (Promega), and luciferase activities of cell extracts were measured with the use of the Dual luciferase assay system (Promega) according to manufacturer's instructions. luciferase activity was measured and normalized for transfection efficiency with Renilla luciferase activity.

### Quantitative telomerase detection

The telomerase activity was measured byusingQuantitative Telomerase Detection Kit (MT3010) according to manufacturer's instructions (US Biomax, Inc). In brief, Resuspend the cell pellet in 200 μl of 1× Lysis Buffer /10^5^-10^6^ cells. Incubate the suspension on ice for 30 minutes. Spin the sample in a microcentrifuge at 12,000x g for 30 minutes at 4°C. Transfer 160 μl of the supernatant into a fresh tube and determine the protein concentration. Mix the 2×master mix thoroughly and dispense appropriate volumes into PCR thin-wall PCR plates. Add 1 μl of test extract, heat-inactivated extracts or template controls to the individual PCR tubes containing the master mix PCR Initial 10 min 95°C HotActivited Tag DNA Polymerase. Activation Step is activated by this heating step 3 -step cycling:Denaturation 30s 95°C;Annealing 30 s 60°C;Extention 30 s 72°C. Cycle number 40 cycles Cycle. The PCR Quantification screen is displayed during the PCR run and presents data as they are being collected in real time. Collect the threshould cycle or CT value after cycles finished. The threshould cycle is the cycle at which a statistically significant increase in Δ Rn is first detected. Threshold is defined as the average standard deviation of Rn for the early cycles, multiplied by an adjustable factor. A standard curve was generated using the reading of the threshold (CT) of Real-Time PCR.

### Telomere length assay

Telomere length assay using Telo TAGGG PCR ELISApuls kit according to manufacturer's instructions (Roche). A standard curve is established by dilution of known quantities of a synthesised 84 mer oligonucleotide containing only TTAGGG repeats.

### Nuclear run on assay

Nuclear run-on was performed by supplying biotin-probe to nuclei, and labeled transcripts were bound to streptavidin-coated streptavidin-agarose Resin [50] The cells are chilled, and the membranes are permeabilized or lysed. The nuclei are then incubated for a short time at 37°C in the presence of nucleoside triphosphates (NTPs) and biotin labeled probe. The number of nascent transcripts on the gene at the time of chilling is thought to be proportional to the frequency of transcription initiation. To determine the relative number of nascent transcripts in each sample, the biotin labeled RNA is purified and hybridized to a membrane containing immobilized DNA from the gene of interest. The amount of biotin activity that hybridizes to the membrane is approximately proportional to the number of nascent transcripts.

### Cells proliferation CCK8 Assay

Cells were synchronized in G0 phase by serum deprivation and then released from growth arrest by reexposure to serum, and then cells were grown in complete medium for assay. The cell proliferation reagent CCK8 is purchased from Roch and the operation according to the manufacturer instruction.

### Soft agar colony formation capacity assay

2 × 10^2^ cells were plated on a 6 well plate containing 0.5% (lower) and 0.35% (upper) double layer soft-agar. The 6 well plates were incubated at 37°C in humidified incubator for 14 days. The cells were fed 1-2 times per week with cell culture media (DMEM). Soft-agar colonies on the 6 well plates were stained with 0.5 ml of 0.05% Crystal Violet for more than 1 hour and the colonies were counted.

### BrdU staining

70-80% confluent cells were cultured for 24 hour before treatment with BrdU (Roche) for 4 hours. Immunofluorescent staining with an anti-BrdU antibody was performed according to the manufacturer's instructions (Becton Dickinson). BrdU positive cells from ten random chosen fields of at least three independent samples were counted.

### Xenograft transplantation *in vivo*

TheFour-weeks athymic Balb/C mouse was injected the liver cancer cells at the armpit area subcutaneously. The mice were observed over 4 weeks, and then sacrificed to recover the tumors. The wet weight of each tumor was determined for each mouse. A portion of each tumor was fixed in 4% paraformaldehyde and embedded in paraffin for histological hematoxylin-eosin (HE) staining. The use of mice for this work was reviewed and approved by the institutional animal care and use committee in accordance with China national institutes of health guidelines.
